# Evaluation of a longitudinal digital citizen science initiative to understand the impact of culture on Indigenous youth mental health: Findings from a quasi-experimental qualitative study

**DOI:** 10.1371/journal.pone.0294234

**Published:** 2023-12-21

**Authors:** Susannah Walker, Prasanna Kannan, Jasmin Bhawra, Tarun Reddy Katapally

**Affiliations:** 1 Johnson Shoyama Graduate School of Public Policy, University of Regina, Regina, Canada; 2 CHANGE Research Lab, School of Occupational and Public Health, Faculty of Community Services, Toronto Metropolitan University, Toronto, Ontario, Canada; 3 DEPtH Lab, School of Health Studies, Faculty of Health Sciences, Western University, London, Ontario, Canada; 4 Department of Epidemiology and Biostatistics, Schulich School of Medicine and Dentistry, Western University, London, Ontario, Canada; 5 Children’s Health Research Institute, Lawson Health Research Institute, London, Ontario, Canada; National Sun Yat-sen University, TAIWAN

## Abstract

**Background:**

Indigenous youth in settler nations are susceptible to poor mental health due to complex intergenerational systemic inequities. Research has shown benefits of cultural connectedness for improving mental health; however, there are few studies which have evaluated the impact of culturally relevant mental health interventions, particularly among Indigenous youth. The purpose of this study is to assess the impact of a culturally-responsive, land-based, active living initiative on the mental health of Indigenous youth.

**Methods:**

This quasi-experimental qualitative study is part of Smart Indigenous Youth (SIY), a mixed-methods 5-year longitudinal digital citizen science initiative. SIY embeds culturally responsive, land-based active living programs into the curricula of high schools in rural Indigenous communities in the western Canadian province of Saskatchewan. In year-1 (Winter 2019), 76 Indigenous youth citizen scientists (13–18 years) from 2 schools participated in the study. At the beginning of the term, each school initiated separate 4-month land-based active living programs specific to their culture, community, geography, and language (Cree and Saulteaux). Before and after the term, focus groups were conducted with the 2 Youth Citizen Scientist Councils, which included students from both participating schools. This study includes data from focus groups of one participating school, with 11 youth citizen scientists (5 boys, 6 girls). Focus group data were transcribed and analyzed by two independent reviewers using Nvivo to identify themes and subthemes. Both reviewers discussed their thematic analysis to reach consensus about final findings.

**Results:**

Baseline focus group analyses (before land-based programming) revealed themes demonstrating the importance of Indigenous culture, identity, history, and language. Youth emphasized the impact of loss of language and culture, the importance of being a helper, and the necessity of intergenerational knowledge transfer. Follow-up focus group analyses (post land-based programming) indicated that cultural school programming led to students expressing positive mental health benefits, increased interest in ceremonies, increased participation in physical activity, and greater knowledge of culture, identity, and ceremonial protocol.

**Conclusions:**

This novel qualitative quasi-experimental study offers a window into the future of upstream interventions in partnership with Indigenous communities, where Indigenous youth can be engaged in real-time via their digital devices, while participating in culturally-sensitive, land-based school programming that promotes culture, identity, and mental health.

## 1. Background

In Canada, Indigenous Peoples consist of three groups: First Nations, Inuit, and Métis. The discriminatory categorization of Indigenous Peoples in Canada is a complex subject, which could be perused in Smylie and Allan’s report, “First Peoples, Second Class Treatment” [1]. Many First Nations people in Canada live on reserves, which are pieces of land allotted to First Nation bands in Canada under the Indian Act, where First Nation band members have the right to live, and band administrative and political structures are located. First Nations do not have title to reserve lands, which are held in trust for bands by the British Crown [2].

Historical and intergenerational trauma have contributed to greater health issues among many Indigenous communities, with Indigenous youth in Canada having suicide rates two to three times higher than the national average [3]. Some studies have reported suicide rates as high as five to six times the national average [4]. Connected to these issues of mental health are culture loss, which includes both tangible losses of language, tradition and customs, and historical trauma. This complex phenomenon is in turn connected to “depression, self-destructive behaviour, suicidal ideation and attempts, anxiety, low self-esteem, and self-medicating to try and hide painful emotions” [5]. Other issues that disproportionately affect Indigenous youth include gang involvement, lack of educational attainment, and poor health, including teen pregnancy, sexually transmitted infections [6] and lower rates of quality physical health [7].

Globally, youth mental health is a large burden on the healthcare system. Due to poor mental health, youth are at risk for outcomes such as “lower educational achievements, substance abuse, violence, and poor reproductive and sexual health” [8], and youth needs for mental health treatment and care are not met, even in high income countries [8]. Youth have the highest incidence of mental health and addiction concerns [9], and the current system of care has not been successful in addressing these gaps. There is a need for new approaches to mental health that incorporate the influence of environment on youth mental health, which also account for societal changes such as economic inequality and digital technology advances [10]. Given the dire global context for youth mental health, Indigenous youth experience the cumulation of multiple marginalized statuses, due to the historical oppression and inequalities faced by Indigenous communities in Canada.

Many reports have been commissioned to confront and offer solutions to these problems; one important report on youth suicide in 2018 from the Federation of Sovereign Indigenous Nations, which represents 74 First Nation communities in Saskatchewan, noted that Indigenous people have been overrepresented in suicide deaths since 1978 [11] and identified the need for comprehensive cultural teachings [11]. In another report from the Saskatchewan Children and Youth Advocate Office after a tragic shooting in the small Dene community of La Loche, Saskatchewan, Canada, youth were asked what they needed to change in their community, and a key response was the renewal of culture [12]. These reports, along with more comprehensive national reports such as the Royal Commission on Aboriginal Peoples report in 1996 which described the need for a renewed relationship between Indigenous people and their dismal health and economic outcomes as a result of colonization, the Truth and Reconciliation Commission report which recommended numerous Calls to Action to repair harms from the residential schools, as well as health reports on the impact of trauma and historical oppression [13], highlight that the key recommendation for prevention of suicide and amelioration of mental health issues is connecting youth with their culture.

Recent research [14] delineated the impact of cultural knowledge on improving mental health for Indigenous youth and the protective effect of culture. For instance, Indigenous youth who report greater cultural connectedness tend to have better health outcomes, and culturally-based interventions have shown promise for Indigenous youth in Canada [15], Australia [16] and the United States [5]. Moreover, the offering of traditional school programming in language and culture under the leadership of school personnel who speak and understand Indigenous languages can significantly increase engagement of Indigenous youth [17].

School-based activity programs, including physical activity, sport, or recreational activities, are beneficial for the holistic development of Indigenous youth. Such programs not only provide benefits in terms of mental health, but also improve perceived spiritual health. For example, evidence indicates that cultural activities such as powwows, prayers and teachings from Elders significantly enhance youth spiritual wellbeing. Activities such as hunting and fishing have potential to strengthen youth connections to the land and support the development of relationships in Indigenous communities, including family and community member support [18].

Sports may be considered as one of the “most salient mediums for recapturing spirits” among Indigenous people in Canada [18]. Particularly, participating in traditional games enhances the sport experiences of Indigenous youth by promoting cultural pride, interaction with Elders, connection with land, and development of positive personal characteristics [19]. Such engagement of Indigenous youth in school activity programs is dependent on participation of Indigenous educators, support on campus, and in the community, including Elders and counselors [20].

Evidence from Australia indicates numerous health benefits of youth participation in cultural activities [16]. Additionally, when these interventions happen earlier in life, children can display more positive developmental outcomes [16], which provides a basis for ensuring greater access to cultural programming in schools to equalize access. Considering the challenging outcomes for Indigenous youth and the benefits of culture, this study addresses a key gap in existing literature–the impact of holistic, land-based cultural programming on Indigenous youth mental health. This study is part of the larger Smart Indigenous Youth (SIY) initiative, which embeds culturally-responsive, land-based, active living programs into Indigenous school curricula to improve youth mental health and address gaps in research on culturally appropriate, land-based and innovative technological solutions. As part of the SIY initiative, this study aimed to identify the influence of a culturally appropriate land-based school program on Indigenous youth mental health by assessing changes between pre- and post-intervention.

## 2. Methods

### 2.1. Smart indigenous youth initiative

SIY is a mixed-methods 5-year longitudinal citizen science initiative that embeds culturally responsive, land-based active living programs into school curricula. SIY is part of the Smart Platform, a citizen science and mobile health initiative for ethical engagement, integrated knowledge translation, and policy and real-time interventions [21]. The Smart Platform uses digital citizen science to co-create knowledge with all participants [22].

SIY uses a Two-Eyed Seeing approach to work with, and alongside Indigenous communities. Two-Eyed Seeing, conceptualized by Mi’kmaq Elder Albert Marshall, notes that “there are diverse understandings of the world and that by acknowledging and respecting a diversity of perspectives (without perpetuating the dominance of one over another) we can build an understanding of health that lends itself to dealing with some of the most pressing health issues facing Indigenous peoples and communities” [23]. SIY integrates Traditional Knowledge and digital citizen science through community-based participatory research approaches [21, 22] to address health issues facing Indigenous peoples and communities” [23]. This approach involves community members at each step of the research process, seeking to build equity into academic projects [24]. Collaboration is a critical component, as well as conducting research that has tangible and relevant benefits to Indigenous communities. Community-based participatory research action seeks to give to communities instead of simply taking data and information [24].

Extensive preparation was done prior to beginning the SIY project, including seeking guidance and input from Elders and knowledge keepers, building relationships with school personnel, and following respectful cultural protocol such as the gifting of tobacco to Elders and knowledge keepers. Before the implementation of SIY, our team built strong partnerships with the communities based on equity, respect, and ownership. This partnership exemplifies not only study co-conceptualization and co-creation of knowledge, but also Indigenous ownership of data and integrated knowledge translation. School personnel who became stakeholders in the project had multiple points of input into the project’s formation and roll-out in the schools through meetings with SIY personnel, and were able to read, offer revisions and approve written articles about the SIY Project as the project progressed. All youth contributing to SIY are citizen scientists, who engage with the research team at the beginning, during, and end of each school term. This engagement is governed by the Youth Citizen Scientist Advisory Council consisting of students from the participating schools. Additionally, the SIY Project has a results-driven approach, which aims to share data, resources, and knowledge with the schools to leverage results for changes in curricula to benefit Indigenous students.

### 2.2 Design

A quasi-experimental study was conducted with 76 Indigenous youth citizen scientists (13–18 years) in year-1 (Winter school term in 2019), with youth from two schools. At the beginning of the term, each school initiated separate four-month land-based active living programs that were specific to their culture, community, geography, and language (Cree and Saulteaux). Before and after the term, focus groups were conducted with two separate Youth Citizen Scientist Councils, one from each participating school. The Youth Citizen Scientist (YCS) Councils consist of youth who participated in the larger project and agreed to participate in the focus groups. Moreover, during the 4-month intervention period from January to May 2019, YCSs engaged with researchers in real-time using their own smartphones via a custom-built application to provide their perceptions of the initiative [22]. This quasi-experimental study includes data from the pre- and post-intervention focus groups of one of the participating schools. The qualitative data collected complements the larger picture from quantitative and qualitative data obtained through the overall Smart Platform study [21, 22]. These focus groups were conducted with 11 youth who formed the Youth Citizen Scientist Advisory Council (5 boys, 6 girls). While the sample size (n = 11) can be viewed as small, research from Hennick and Kaiser [25], Guest et al. [26], Mishra [27], and Mason [28] notes that sample size for focus groups can approach saturation of themes with eight to ten participants, as our qualitative data is not seeking to prove a hypothesis, but rather to identify relevant and unique themes from focus group discussions. Before any data were collected ethics approval was obtained from the Research Ethics Boards of Universities of Regina and Saskatchewan through a synchronized review protocol (REB # 2017–29)” [22]. More importantly, every youth citizen scientist provided informed consent before both focus groups, where they had the opportunity to ask questions about data privacy, security, and sovereignty. They were advised that their participation was completely voluntary. After taking permission from the youth, focus groups were recorded and transcribed by the SIY research team. The research team was in constant communication with the school principal and educators to prepare for any potential barriers.

### 2.3 Procedures

To ensure respectful data collection, this study followed relevant articles outlined in Chapter 9 of the Tri-Council Policy Statement 2, the Canadian Institutes of Health Research guidelines for working with Indigenous Peoples, and the principles of Ownership, Control, Access, and Possession (OCAP®) from the First Nations Information Governance Centre [29].

The Tri-Council Policy Statement on ethical research with Indigenous communities outlines a number of important articles to conduct appropriate and respectful research that are applicable to the SIY Project, including Article 9.12: Collaborative Research, Article 9.13: Mutual Benefits in Research, Article 9.15: Recognition of the Role of Elders and Other Knowledge Holders, and Article 9.16: Privacy and Confidentiality [30]. SIY also follows Canadian Institutes of Health Research guidelines for creating cultural safety, which is “a participant-centred approach that encourages self-reflexivity among health researchers and practitioners… [and] requires building trust with Indigenous Peoples and communities in the conduct of research” [31]. To respect OCAP®, ownership of data by the Indigenous communities is a key part of the SIY Project [29].

### 2.4 Culturally responsive, land-based intervention

The land-based intervention component of the program was created and administered by school personnel in partnership with Elders, knowledge keepers, and our research team. The focus of the intervention was holistic wellness, including physical, mental, emotional, and spiritual health. Land-based interventions include ceremonial and cultural activities which seek to foster a connection or re-connection to the land. They can encompass a wide range of activities, such as traditional food practices, Indigenous birth practices, language and cultural camps, and Elders and Knowledge Keepers sharing about coming of age ceremonies, ethical practices about being on the land and co-existing in good relation with the natural world [32].

**Fig 1:**
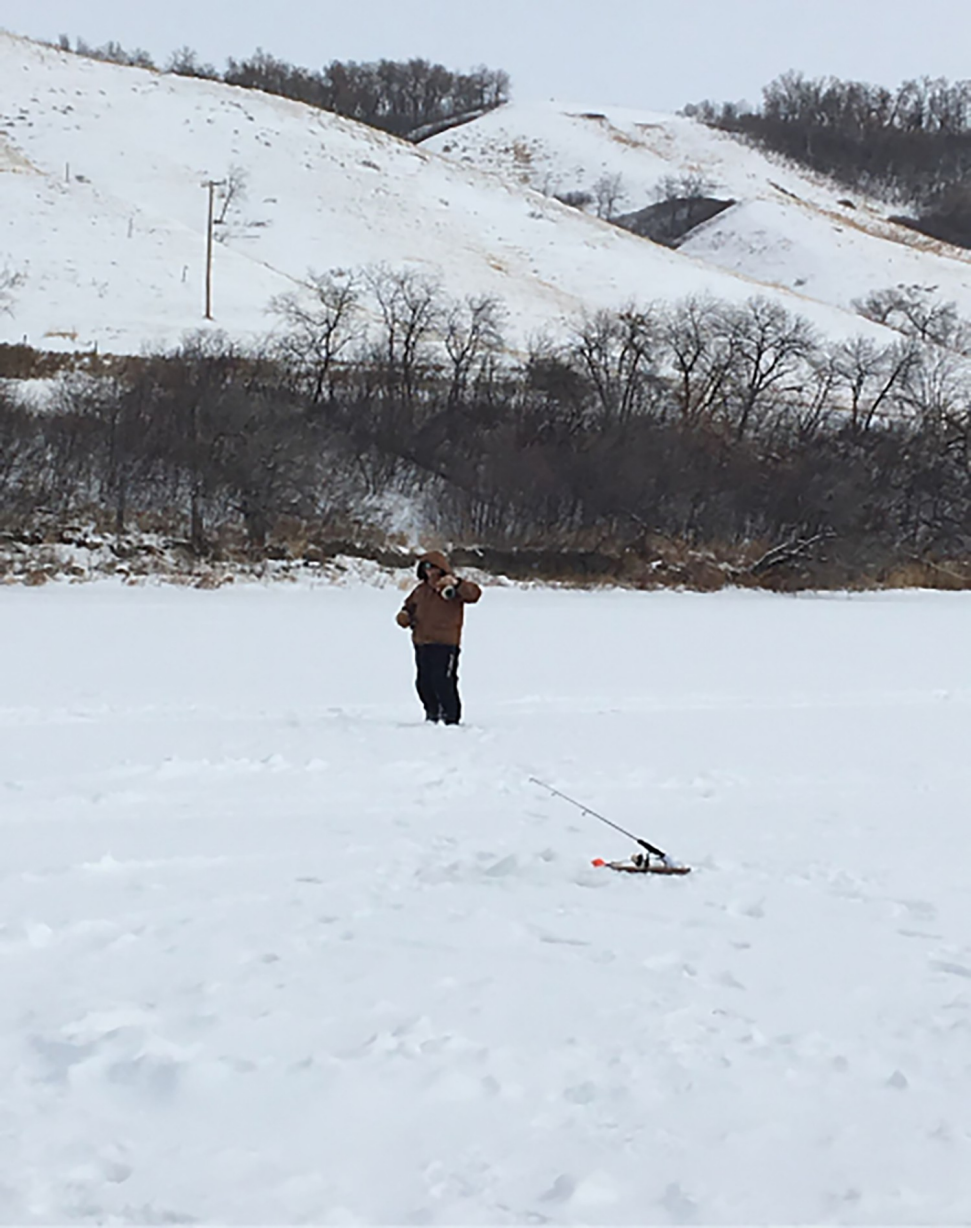


**Fig 2:**
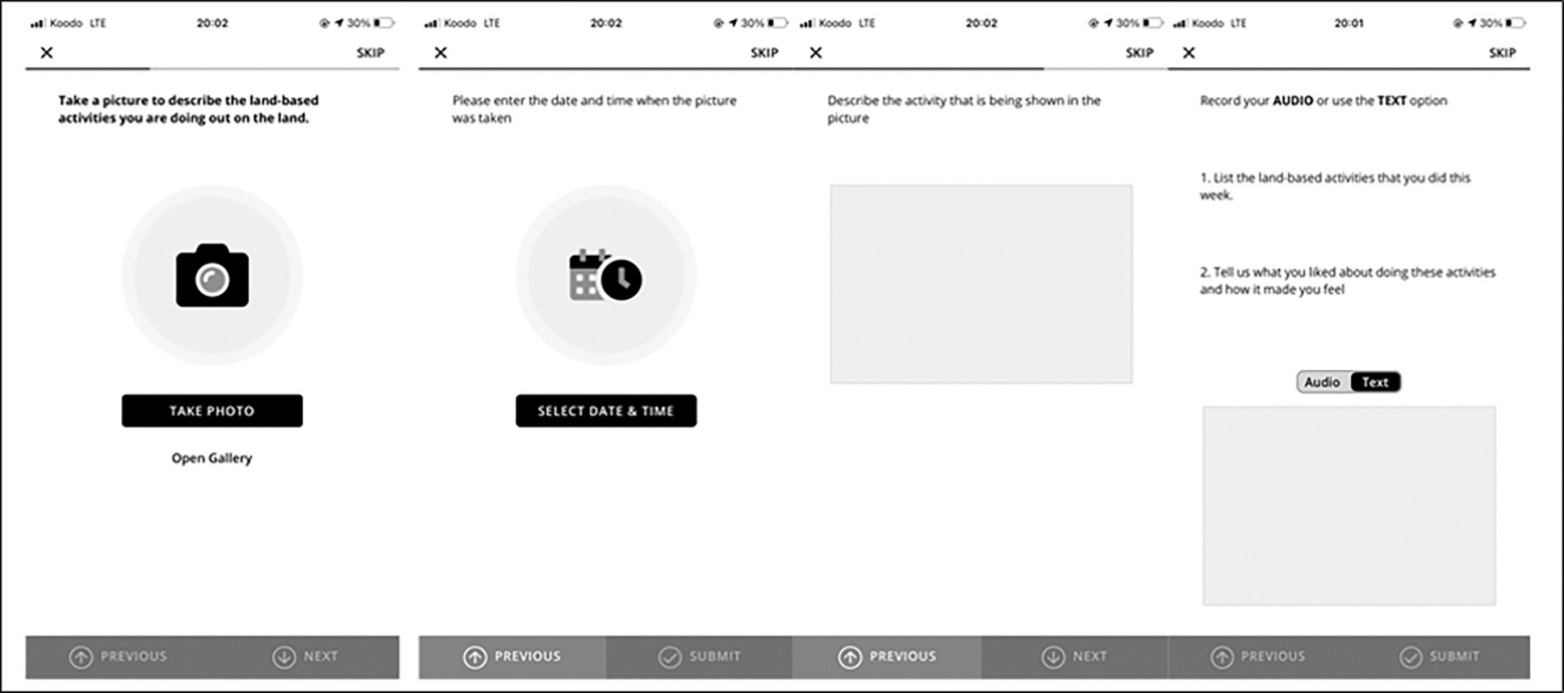


The school in Saskatchewan where the land-based intervention was held will be referred to as School 1 to preserve its anonymity. School 1 chose to implement a combination of sports and cultural activities, including sweats, pipe ceremonies, Elder and knowledge keepers guest talks, art activities including moosehair embroidery and quill work, feasts, dancing, ice fishing, medicine walks (walks in nature with Elders and knowledge keepers to collect sacred medicines such as sage and sweetgrass) and plant identification using Cree language. The activities also included community events such as Flower Day (a day to honour ancestors), and a lodge with ceremonies such as the Rain Dance and Sun Dance. Activities were offered approximately three times per month over the winter term. Students were encouraged to participate in all activities but attendance was not mandatory. Educators, administrators, Elders, artists, family members and cultural teachers participated in the programming. Youth who participated in the land-based activities were asked to take pictures via the custom-built smartphone app. Questions were pushed in real time to allow youth to capture photos and audio during the land-based activities (Figs 1 and 2). These ecological momentary assessments enabled the youth citizen scientists to provide critical information about their perception and engagement with the intervention.

### 2.5 Qualitative data collection

The focus groups with the Youth Citizen Scientist Advisory Council (11 students aged 13–18 years) were conducted in two rounds of talking circles to respect Indigenous ways of knowing: two rounds of one circle for the boys (n = 5), and two rounds of one circle for the girls (n = 6). The separate circles were formed based on the feedback from the school administrators, educators, Elders, and the students themselves. The design also ensured that the same students participated in pre- and post-intervention focus groups. Focus groups were facilitated by two Indigenous graduate students and included a small incentive as a gift for the student participants. Incentives included toques, frisbees, and jump ropes to encourage physical activity. Indigenous facilitators began the circle with a smudge and a prayer, followed by an explanation of the privacy and participation guidelines for the circle (i.e., option to skip questions if uncomfortable), and participants passed around and held a small stone for participants as they spoke. The smudge was left burning in the centre of the circle for participants’ comfort and in respect to the emotional topics of identity and perception of mental health. One facilitator asked the questions and the other took notes for the students to see on a large whiteboard. The circle was closed by shaking hands.

Pre- and post-intervention semi-structured focus group guides (S1 Appendix) were developed collaboratively with the school to capture the impact of the culturally-responsive, land-based, active living program on youth perception of mental health. Based on the type of land-based activities that the school implemented, the post-intervention focus group template was tailored to capture changes in youth behaviours and outcomes. Mental health supports were available through the school in the event that students experienced any emotional difficulties from their participation in the focus groups.

### 2.6 Data analysis

Focus groups were audio-recorded, and data were anonymized during transcription into Word documents. The Word documents were then uploaded into Nvivo 12 to thematically code and analyze data. Two researchers completed data analysis using the word frequency query tool in Nvivo. The most common words and phrases were used to create a number of ‘nodes’ or categories. The researchers then reviewed the nodes for commonalities and combined them into four overarching nodes, i.e., themes. These four ‘parent’ nodes were then reviewed and the researchers created ‘child nodes’ or sub-themes within each theme. By reading and rereading the data, the researchers selected the most relevant and impactful quotations from focus group participants to illustrate each sub-theme. A final review was completed by both researchers to look for further patterns or areas of overlap among the themes before selecting the final themes and sub-themes. Each researcher completed the thematic coding independently, both using Nvivo and manually, and then compared their findings before coming to a consensus. A third researcher reviewed the transcripts, thematic coding, and themes and sub-themes for accuracy and coherence before validating the consensus of the first two researchers. This was a collaborative process between researchers to find greater meaning, objectivity, and understanding from the transcript data.

## 3. Results

**Fig 3:**
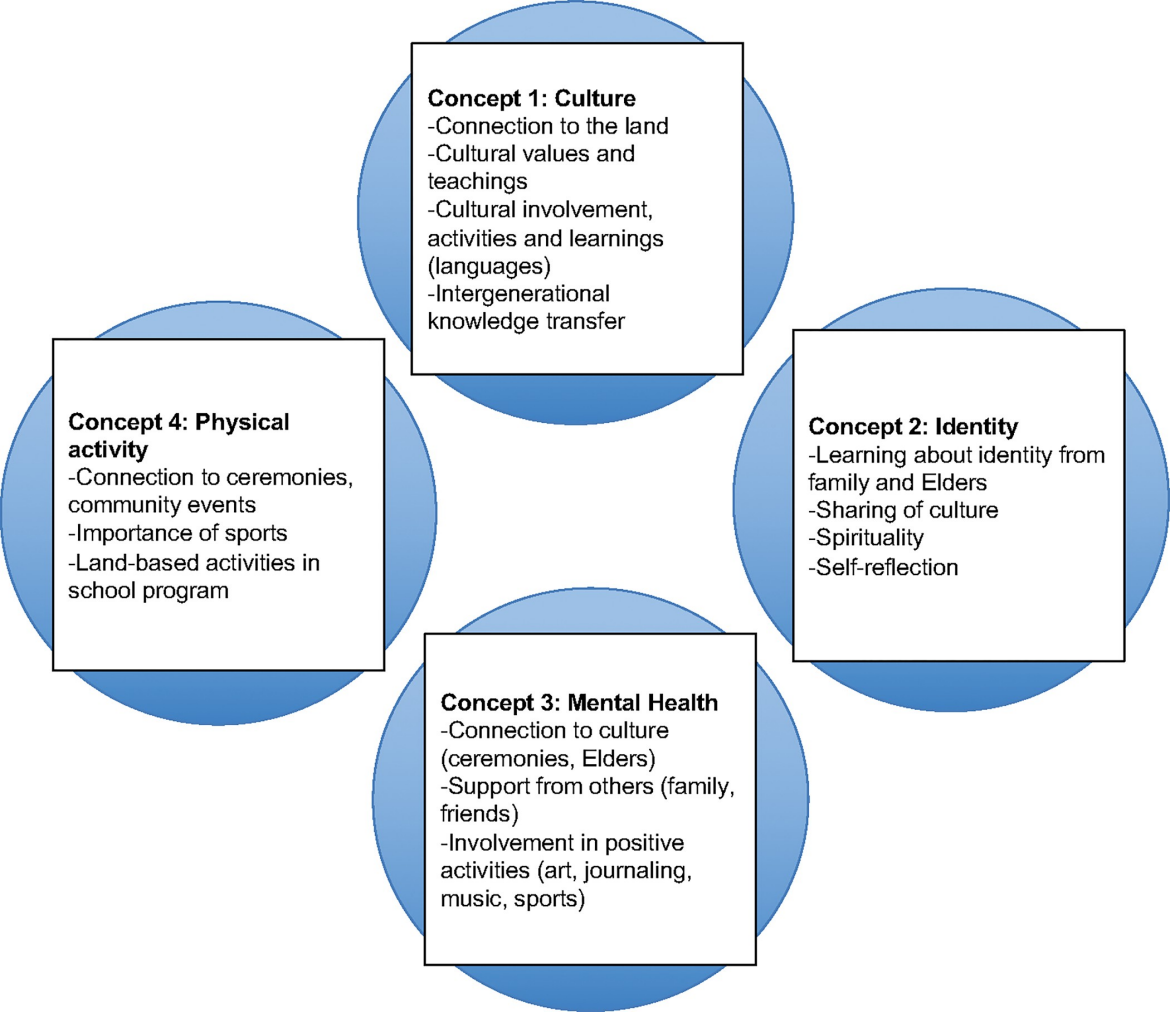


The four main themes (Fig 3) that emerged from focus group discussions with youth citizen scientists were: Indigenous culture, identity, mental health and physical activity, with youth emphasizing the impact of the loss of language and culture, the importance of being helpers, and the necessity of cultural connectedness across generations. Intergenerational transmission of knowledge, both from Elders and to younger generations, was also noted.

In particular, post-intervention focus group analyses showed that through participation in the land-based school programing, students expressed increased interest in ceremonies, knowledge of culture, identity, and ceremonial protocol. There were a number of overlapping themes across boys and girls, including the impact of loss of culture and tradition, importance of intergenerational transmission of knowledge, value of peer support, need for sports and physical activity, and the positive impact of the culturally-responsive, land-based, active living school program.

### 3.1 Themes and sub-themes

#### Concept 1: Culture: Changes between pre and post intervention

At baseline (i.e., pre-intervention), youth citizen scientists were asked about the importance of culture and how they defined culture. In the follow-up focus groups, we discussed if and how the land-based programming influenced their understanding of culture. The overall concept of culture elicited a number of sub-themes, including connection to the land, cultural involvement, cultural values and teachings, cultural activities and learnings (languages), and intergenerational knowledge transfer.

Multiple male participants expressed that they stayed involved in their culture by:

“…just helping out at ceremonies and stuff. Giveaways, feasts and stuff. Round dances” (Citizen Scientist 2, p. 4) and by “helping the Elders get the trees and all that” (Citizen Scientist 3, p. 4).

Others shared that they enjoyed:

“Helping out Elders with cultural stuff… helping out at ceremonies” (Citizen Scientist 2, p. 4–5) “and in the community by “helping Elders cut trees for their wood stoves” (Citizen Scientist 4, p. 5).

The role of a helper for ceremonies is traditionally a male role and it was evident that this resonated with the boys’ group.

When asked about the impact of the land-based program and how it might altered their perception of culture, post-intervention, respect and gratitude for the land were mentioned by students in the girls’ as a change from participating in the land-based program. Citizen Scientist 3 noted that for her, culture means:

“…just how to respect the land and what we’re given and to be very grateful about the life that we have” (Citizen Scientist 3, p. 2).

Citizen Scientist 4 also noted that the land-based programs:

“[It] made me think that the–like, I started not to litter that much because I think about it and I think about how the world is so important to us. And I think about how this world is going to go in trash pretty soon trying to like–just want people to take care of it more. Whenever I–like my friends throw something on the garbage I’m like pick it up! And put it in my bag because I’m going to put it in the garbage later. And that’s what yeah–I just care about what’s happening now and we should take care of it and we should respect it” (Citizen Scientist 4, p. 7).

Keeping culture going was identified as very important in the female follow-up focus group. One participant noted that she:

“…learned that our culture plays a big role in our future life and in our present life” (Citizen Scientist 3, p. 2).

Generational connections were identified by participants as well; one participant remarked that:

“most of those activities are going to last a long time, a lot of generations because people like it and we should pass it on too to keep it going” (Citizen Scientist 4, p. 2).

Another expressed that she was:

“…really thankful that it’s actually getting passed down to us because it means a lot to like know what our people did in the past and that it’s still present to this day” (Citizen Scientist 6, p. 3).

Others expressed their own sense of responsibility for cultural knowledge by saying:

“We just gotta keep it going, make it known and it’s very important to have it” (Citizen Scientist 7, p. 3) and “I want to pass it on to other people when they’re younger and I want to teach it properly” (Citizen Scientist 4, p. 4).

Additionally, a key change identified in the female follow-up focus group was having acquired more cultural knowledge from the land-based program. One participant said:

“I feel like it gives it more meaning and I know what’s going on. And I know how to react to different things” (Citizen Scientist 3, p. 4).

Another participant expressed that culture:

“is important to me because from not knowing about the, um, activities made it more important because I know how they work and how to like respect them and respect other people around me” (Citizen Scientist 5, p. 4).

Lastly, another participant expressed that:

“Culture is important to me because it actually tells me who I am and who I want to be and just gives me more knowledge to pass down. Uh, it also helps with just yourself, what you believe in and like just how you see yourself and other people and the world” (Citizen Scientist 7, p. 4).

These changes are important because they could help reduce barriers for Indigenous youth to participate in cultural activities, and based on the changes observed post-intervention, the following sub-themes have been identified: connection to the land, cultural involvement, activities, and learnings, cultural values and teachings, and intergenerational knowledge transfer:

*Connection to the land*. Female students identified female teachings (moontime or menstrual cycle teachings), the connection to Mother Earth, and the value of walking around on the land as an enjoyable activity. Respect and gratitude for the land were also mentioned by students in the follow-up focus group as a change from participating in the land-based program.

*Cultural involvement*, *activities*, *and learnings*. Both male and female students mentioned their involvement in ceremonies, such as sweats, round dances, powwows, shaking tent, rain dances, sundances, tipi teachings, and dancing powwow. Students also discussed being involved with cultural activities through school, including traditional sports, daily smudging, beading, ribbon skirt making, sage picking and school-organized sweats. The boys mentioned specifically being culturally involved by being a helper, both at ceremonies and with Elders in the community.

*Cultural values and teachings*. Helping Elders, listening to stories, respecting Mother Earth, and knowing the protocol for ceremonies were all mentioned as cultural values and teachings. Passing on knowledge was also a key aspect:

“So, I can use it in future purposes and also to like, teach the younger ones about like what we learned” (Citizen Scientist 3, p. 2)

Having a positive mind when participating in ceremony was identified as important:

“Like how you feel like when you’re going into a ceremony, it will change. Like if you are feeling bad or mad about yourself, you can go into a sweat or other kind of ceremonies and–or even a powwow and just start dancing or something and you’ll feel better about yourself.” (Citizen Scientist, p. 8)

as was knowing the language:

•“Keep learning about it and keep trying to learn your language and stuff like that, because if not, then our language and traditions would no longer be, I guess” (Citizen Scientist 5, p. 2)

*Intergenerational knowledge transfer*. Students identified the loss of culture and language due to residential schools as something that was concerning to them, and the importance both of teaching the younger generations about culture and traditions, and taking the time to learn from Elders. One female student shared that her culture was important to her:

“…because, well, usually like back then, the kids would be born into that kind of lifestyle and like after all residential schools and all that stuff happened, Natives kind of lost their language and their traditions. And I find it like really important to keep it, keep learning about it and keep trying to learn your language and stuff like that, because if not, then our language and traditions would no longer be, I guess” (Citizen Scientist 5, p. 2).

#### Concept 2: Identity: Changes between pre and post intervention

In the focus groups, students were asked about how they learned about their identity. One participant expressed that she was taught that her:

“identity was in [my] community, at school and [from my] parents and grandparents, pretty much the people that surround me” (Citizen Scientist 4, p. 3).

The Cree language program at the school was mentioned as a key aspect of learning identity, especially since one participant shared that her family didn’t speak the language at home:

“I learnt it in kindergarten. [Laughter] with all our–with my teachers. Well she is still here today. When I was in kindergarten, my mom really never talked about it or my parents, because they don’t really do that stuff. So, yeah mostly at school” (Citizen Scientist 3, p. 3).

When asked about changes in their identity from participating in the land-based program, the girls’ follow-up focus group identified two important changes around the theme of identity: feelings of goodness and changed feelings about self. One participant described how she viewed the change:

“I do feel a change like I feel that my spirit is fed… like with goodness” (Citizen Scientist 3, p. 5).

Another participant expressed that after participating in cultural activities that:

“I feel good about myself because I always feel good after” (Citizen Scientist 4, p. 5).

Another key change that was noted by other participants was changed feelings about self. One participant said that

“it kind of changed how I feel about myself” (Citizen Scientist 5, p. 5).

Another said that her worldview had changed, expressing that:

“there will be a change in the world and how you see it, and then how you see yourself” (Citizen Scientist 6, p. 6).

These findings highlight the connection between positive self-concept and mental health.

A number of sub-themes emerged: learning about identity from family and Elders, sharing of culture, spirituality, and self-reflection.

*Learning about identity from family and Elders*. Participants mentioned learning their identity through family members, especially parents and grandparents, through language and through school.

*Sharing of culture*. Sharing of culture with non-Indigenous people was identified as a benefit of the school program:

“[The cultural program] is good because it just brings like more information to our school and us sharing who we are to people who come to our school and what they teach us, like say if they come to our school and teach us something or tell us something and we experience it, it’s not only them teaching us, it’s us teaching them.” (Citizen Scientist 6, p. 10)

Sharing culture with younger people, including siblings, was identified as important.

•“And um, helping our younger siblings showing that you have to do, like dancing. Like I was dancing when I was little and now my sister and now my brother’s dancing” (Citizen Scientist 3, p. 5)

*Spirituality*. Identity was connected to spirituality through the participation in ceremony such as sundance, raindance, speaking Cree language, and doing sweats:

“I grew up kind of in kind of like a traditional family. And then like things happened and I stopped learning about traditional things and stuff like that. But once I got a little bit older, I think I was like 11 or 12, I started to go more to sweats and rain dances and more different kinds of ceremonies” (Citizen Scientist, p. 2)

“I just like dancing powwow, listening to Elders’ stories and teachings behind going to like the ceremony I’m going to or something like that. Hearing how or what happened during this whole ceremony and stuff like that, really makes me want to go especially sweats. I really enjoy going to sweats and stuff like that. Mostly the Elders motivate me to go, usually” (Citizen Scientist 6, p. 9)

Participants also talked about learning cultural identities through elders and teachers.

“Well, it started off at home. When I was little, I would hear people speaking Cree like my moshum and all that stuff and then I would respect it because they were older and they had more knowledge, I guess” (Citizen Scientist 6, p. 3)

•“And then, when I came to school, I think I was in Grade One was the first time I heard someone say the Cree prayer like on the intercom. And I kind of started laughing and I remember it. And then as I got older, I’m pretty sure I was in grade two or three is when I started taking it more serious” (Citizen Scientist 6, p. 3)

*Self-reflection*. Participants discussed identity as something that shows who they are. Identity comes from knowing who they are and where they come from and so on which is transformed from parents and grandparents:

“It shows you who you are, and also just basically shows who you are and what you do and why you do it and what you’re going to be” (Citizen Scientist 2, p. 3)

“My–our identity I think it’s really interesting. I actually feel proud to be who I am, and I never see myself any different. My identity is like who I am right now” (Citizen Scientist 3, p. 4)

Identity is also perceived as a way people think, act and respond to people in the community.

“Yeah, I think my identity shows ya, who I am, and it shows other people that don’t know me who I am. And it will like tell a lot just by like how you act and how you react to other things people say” (Citizen Scientist 5, p. 4)

•“Well, I learned my identity was in my community, at school and my parents and grandparents, pretty much the people that surround me” (Citizen Scientist 4, p. 3)

#### Concept 3: Mental health: Changes between pre- and post intervention

Students were asked how they defined the term ‘mental health’ and they shared that mental health meant positive thinking and surrounding themselves with good people. One participant defined mental health as:

“What mental health means to me is to take care of your mind. [Laughter] Just like positive thinking into your mind, not negative thinking. So, listening to music, listening to positive powwow music, it will give you good thoughts” (Citizen Scientist 3, p. 6).

In the girls’ follow-up focus group, students reported a number of changes in perception of mental health as a result of participating in the land-based program, including feeling refreshed/cleansed, feeling a connection to the land, reduction in stress, being grounded in the present, and thinking more positively. One participant noted the positive impact of participating in ceremonies:

“If I do go to sweats, I do feel like more refreshed. And–or like a powwow or a feast, or anything like that, I just feel like refreshed and like, I don’t know. Just feels like–like cleansed, especially after a sweat” (Citizen Scientist 3, p. 7).

Another noted the positive impact of cultural activities on reducing overthinking and stress:

“… because before when I never used to, like, go to any of these things I’d just chill out and like just think about things, and sometimes it gets overwhelming and like, stresses me out and things like that, but when I like go to like, these cultural things, it just like takes all that off my mind, and just like focuses on what’s happening at the moment” (Citizen Scientist 5, p. 8).

Another participant noted how her thinking had changed for the better after taking part in the land-based program:

“[It] changed my perception of mental health a lot actually. Like, things weren’t really as good as they were now, and now that like I’ve been going to them I think more positively and I’m like really glad that our people do have these things and that we get to learn about them” (Citizen Scientist 6, p. 8).

Lastly, one participant spoke of finding more direction for herself as:

“…the programs I went to changed me and this did too, because basically I feel more just interested and humble and more mature with what I want to do. And I feel like that if I keep on doing it then maybe I would get inspiration” (Citizen Scientist 7, p. 8).

The sub-themes that emerged were connection to culture, support from others, and involvement in positive activities.

*Connection to culture*. Students discussed the contribution of ceremonies to positive mental health, including the necessity of participating in ceremony with a positive mind, and the ability of ceremonies such as sweats to transform bad feelings:

“So, basically say if you’re at a ceremony, you may decide to like to feel down about yourself. Then obviously, in our teachings it’s said that if you feel down about yourself, it’s—you will pass it around and everybody will start to really down on theirselves [sic]” (Citizen Scientist 2, p. 11)

The boys in particular mentioned helping Elders in the community and at ceremony as connected to mental health:

“So to me, like some examples on what I do? Ok so I go to ceremonies. I take knowledge from older people. I do sports and I… basically it means to me like, just what I do to help myself” (Citizen Scientist 2, p. 9–10)

*Support from others*. Students mentioned relying on others for support with their mental health, including talking about feelings and issues with family members and friends:

•“I just I surround myself with good people” (Citizen Scientist 4, p. 6)

•“if I’m like feeling down or something, I’ll just talk to my mom or one of my friends about how I am feeling everything” (Citizen Scientist 5, p. 6)

•“And I hang out with people who make me feel good about myself. Ok, I really–I have a low self-esteem, but my friends help me to feel good about myself and stuff like that” (Citizen Scientist 6, p. 7)

*Involvement in positive activities*. Students mentioned journaling, drawing, cultural activities such as beading, and playing sports to deal with thoughts and emotions:

•“I have a book I write in. Well I have like four. I like to write a lot. And so, that’s what I do to like help me and stuff like that” (Citizen Scientist 6, p. 7)

•“Writing down your thoughts because if your mind gets—keep—getting filled up with all your emotions it will just start to feel down about yourselves. So, writing down or just–and then throwing it in the fire later or something. Then, you have a clear mind and you would -you’re not always depressed. So, you can just live happy. Or even drawing what you feel. Drawing helps with me. Um, beading, something to just get your mind off all the bad things” (Citizen Scientist 3, p. 7)

•“… beading and stuff like that. And drawing, like something that is quiet and that you’re by yourself. But you’re also focused on finishing what you are doing.” (Citizen Scientist 6, p. 7)

•“Playing sports” (Citizen Scientist 2, p. 4)

#### Concept 4: Physical activity: Changes pre- and post-intervention

When asked about physical activity, youth discussed the connection between ceremonies and community events, the importance of sports, and the land-based activities in the school program. When asked about changes occurring from participating in the land-based program, the girls’ follow-up focus group identified a number of changes around their feelings about school. Two participants expressed their interest in learning new things through the land-based program and how these programs made them want to be at school more often:

“It does make me want to go to school more. Because obviously it’s an opportunity to learn something new instead of putting the same old stuff in school. [laughter] It’s actually something that’s like fun and interesting and has a real–well not real, but like a cool story behind it and why” (Citizen Scientist 3, p. 9).

The other participant shared her excitement at knowing there were cultural activities planned for school:

“it made me like want to come to school more and it’s like–really awesome I guess, like, just like, [teacher] or someone coming saying, oh there’s going to be a sweat or there’s a pipe ceremony–it just like–I’m like yes! [laughter] Yay! And yeah, it’s just good” (Citizen Scientist 6, p. 10).

Another participant noted that these types of programs increase the profile of the school, saying that:

“I think these programs make our school look better… it feels good” (Citizen Scientist 4, p. 9).

Another participant expressed her enjoyment of learning about culture through school:

“It makes me feel good because when you’re just at home or something, not at school, just nothing to learn about but when you come to school and there’s opportunities to learn something new about your culture it’s kind of–pretty, pretty dope” (Citizen Scientist 5, p. 10).

Based on these observations the following sub-themes were identified: connection to ceremonies, community events, importance of sports, and land-based activities in school program.

*Connection to ceremonies*, *community events*. When asked how people stayed physically active in the community, youth mentioned ceremonies such as sundances and sweats.

“By doing like, just like sports and by going to ceremonies, by doing community things like, say there was a barbeque or something like that, or else like a community feast or something like that. And yeah, that’s about it” (Citizen Scientist 2, p. 5)

“Um I think, like usually, we see like people, like going on walks and stuff like around the hills and stuff and like also like powwows and round dances. And sports, I think sports is a big part of our school too. So, I think that’s one way our people stay active, I guess” (Citizen Scientist 4, p. 6)

•“They still put up sweats” (Citizen Scientist 2, p. 3)

•“The walks where the Sundance is” (Citizen Scientist 2, p. 3)

*Importance of sports*. Students mentioned how much they enjoyed the sports offered at school and the activities associated with sports teams, along with physical education class and gym nights offered at the school:

“Physical education, probably focusing on doing my work and just paying attention, I guess. I like being involved in a bunch of stuff” (Citizen Scientist 2, p. 12)

“I like doing, um [laughter] activities after school, like playing sports, going on sports trips with friends because it takes off all the stuff of my mind” (Citizen Scientist 3, p. 13)

“I like to coming to school for sports and learning about new stuff” (Citizen Scientist 4, p. 13)

“The thing that I like about school is that they involve a lot of people in sports and a lot of them” (Citizen Scientist 5, p. 13)

Community sports events and community sports nights were popular:

“And sports is like a really big thing out here, like baseball. Mostly summer sports and hockey. People really like it out here and mostly sports and walking around and powwows and stuff like that” (Citizen Scientist 6, p. 6)

“And summer games, we try out. All FHQs [File Hills Qu’Appelle nations] tryout and then there is different people. It’s pretty fun. And winter games” (Citizen Scientist 5, p. 7)

*Land-based activities in school program*. Students discussed how much they liked land-based activities such as medicine picking, ceremonies such as sweats, and other cultural activities such as beading and skirt-making.

“I like beading. I wish there was more programs in this school like when we’re—like skirt making (Citizen Scientist 3, p. 13)

“Picking our own sweet grass and sage” (Citizen Scientist 4, p. 8)

“I like coming to school because our school’s like different, like we go to do ceremonies and stuff like that. Like sage picking and stuff… more like that kind of stuff” (Citizen Scientist 6, p. 13)

“We have opportunities to go to sweats. We never been to—I’ve never been to a sweat for a long time. But sometimes they’ll—Alphonse will bring it up, he’ll be like, want to go to a sweat? We all go and it’s a good cleansing for all of us and we come back to school, smelling like sweat. [Laughter] People think it’s jokes, but it’s not” (Citizen Scientist 5, p. 6)

## 4. Discussion

This quasi-experimental qualitative study aimed to identify the association between culture and the mental health perception of Indigenous youth who participated in the land-based active living program that was tailored to their culture, language, and history. The overarching findings showed strong connections between culturally-sensitive, land-based learning and perception of positive mental health among Indigenous youth.

These findings contribute to our understanding of the importance of cultural identity for Indigenous youth to protect them against negative mental health outcomes and provide them with resilience. The findings align with existing evidence that shows that Indigenous youth with connections to their cultural identity reported greater degrees of resilience [11]. Given the positive impact of cultural activities on students’ perception of mental health, it is important for schools to provide Indigenous students with opportunities to participate in ceremonies. For example, providing sweats either on-site or partnering with community Elders to provide opportunities for students to participate would be a culturally-appropriate way to meet this need.

The results also showed the importance and need for land-based activities in potentially improving Indigenous youth holistic health [1, 14, 33–35] i.e., the concept of health as portrayed by Indigenous youth citizen scientists shows the impact of land-based programming on not just physical health, but also mental, emotional and spiritual health. This concept of health aligns with Indigenous ways of knowing, and approach to wellbeing in Indigenous teachings, with the mental, emotional, physical and spiritual needing to be in balance for optimal health [36–39]. For Indigenous youth, a holistic approach to health that includes and fosters cultural connection is considered best practice for mental health and for mental health interventions. The sub-themes of connection to culture (ceremonies, Elders), support from others (family, friends), and involvement in positive activities (art, journaling, music, sports) came directly from asking the Indigenous youth involved how they saw positive mental health and what components were involved. This also aligns with community-based participatory research principles that take a partnering approach to research [24] and work to meet participants where they are at. In essence, the sub-themes under mental health are addressing the social determinants of mental health i.e., the root causes and coping strategies) rather than treating specific mental health issues.

As youth emphasized their interest in the activities that took them out of the classroom to connect with the land and their culture, it is important for schools to consider how they can adapt their curricula to incorporate land-based, active living–an approach that could have an impact on youth irrespective of their identity. Some gender-based differences emerged as boys expressed greater interest in gathering trees to assist Elders in building ceremonial structures, being on the land, helping Elders and being helpers at other community events. Providing opportunities for boys to participate in culture in these roles could be meaningful in facilitating a sense of value and wellbeing, as well as reconnecting to culture to repair damage to Indigenous masculinity through processes of colonization [40–42].

The findings show that land-based activities are of high interest to Indigenous youth and could be used as a natural way to build cultural knowledge and pique youth engagement. Land-based activities and curricula are increasingly popular for connecting Indigenous youth with their culture and identity [43]. However, in our study, we clearly depicted that land-based, active living programing can have a particularly positive impact on perception of Indigenous youth mental health, while enhancing cultural competence, identity, and active living i.e., these concepts are potentially inherently connected together to enhance holistic health. Another avenue of possibility is the connection between our results and shaping Indigenous youth attitudes towards mental health services. If mental health services have holistic, cultural, and land-based components to their programming, Indigenous youth may be more inclined to partake, which could have positive implications for improved overall mental health.

When considering the results, schools emerge as natural sites for cultural and mental health promotion for Indigenous youth. Building cultural programs with trusted teachers and knowledge keepers as a part of school programming for Indigenous youth would be a beneficial addition to on-reserve school curricula, as well as off-reserve schools with high populations of Indigenous youth. Cultural programs could be housed or offered as a part of comprehensive mental health services for students. Youth demonstrated high engagement and interest in cultural activities provided by the school. Some youth shared how they had learned about their Indigenous identity from school activities and teachers. Schools with Indigenous populations and on-reserve schools have a role in increasing cultural knowledge for Indigenous youth. School attendance rates could also be positively impacted. Dockery [16] notes the importance of involving schools in cultural resurgence for Indigenous populations, noting there is “a need for changes in social attitudes and institutional practices to support and celebrate the maintenance of traditional Indigenous cultures” [16].

Dockery [16] also emphasizes that Indigenous “identification with those cultures, particularly within the education system, and the reversal of policies that are predictably contributing to the disappearance of cultural practices, knowledge and languages” [16] can potentially play a key role in Indigenous cultural resurgence–a factor that can positively impact Indigenous youth perception of mental health, as depicted in our findings. Wanting to learn more about teachings, about ceremony, and about language was expressed by a majority of youth. Youth also demonstrated understanding of the scale of loss and the urgency of learning traditional knowledge in order to protect it going forward. Schools providing more opportunities for students to participate in culturally-based programming would help fill this interest expressed by students. Considering the past damages to Indigenous culture from oppression and abuse in the residential school system, providing avenues within the schools to remedy this, along with familial and community efforts, would be of benefit to Indigenous youth.

At a policy level, it would be beneficial for school curricula to be modified to include cultural or land-based programs at all Indigenous schools, with programs for all ages of students. Current programs such as the Cree immersion program in Saskatoon [44] or land-based programs at Bert Fox Community School in Fort Qu’Appelle, Muskeg Lake Cree Nation, or the Buffalo River School, among others, could be used as models [45]. The importance of intergenerational connection was expressed by both girls and boys in this study, including connections between past and present, youth and Elders, and teaching the next generation. Programs that provide avenues for this knowledge transfer between Elders and youth will help to build cultural knowledge, and strengthen identity [46]. This would create stronger connections between youth and their community, in addition to reflecting traditional ways of passing on knowledge [47]. In essence, providing intergenerational programs could have a meaningful impact for youth, both in the school and community setting.

Apart from the imminent need for culturally-sensitive, land-based school programming, a key recommendation from our study is the necessity for systems of care to move to a prevention-oriented, upstream, long-term model with flexibility and innovative approaches. For instance, we operationalized digital citizen science using smartphones of Indigenous youth to engage with them in real-time–this approach has potential to respond to Indigenous youth needs in real-time, an innovation that is particularly useful in remote Indigenous communities [48]. This approach points towards more upstream interventions and fewer reactive systems of care when Indigenous youth are in serious danger. Nevertheless, for the success of digital innovations in Indigenous communities, it is critical to incorporate Two-Eyed Seeing, community-based participatory initiatives.

### 4.1 Strengths and limitations

The strengths of the study are the study method, youth empowerment, and culturally respectful approach, and the holistic health benefits from participating [22]. Youth were empowered as citizen scientists through the focus group talking circle approach, which was collaborative and culturally respectful, and allowed them space to voice their thoughts and reflect on their identity and cultural needs. Focus group discussions have numerous strengths, particularly the ability to obtain rich contextual data which enables us to answer the question of ‘why’ we observe certain behaviours, health outcomes, and relationships between variables. Longitudinal qualitative study design is a major strength of this complex study as it allows us to track and empirically capture the impact of culturally-sensitive, land-based active living programming on Indigenous youth perception of mental health.

Limitations of the study included the sampling strategy and size, compliance and response rate, logistical and technological issues, as well as participant burden. Sampling was not random, as School 1 self-selected to participate in the SIY project. Students were invited by school personnel to participate in the focus groups, and in essence, self-selected themselves as well. While the sample size is a potential limitation, the recruitment strategy was appropriate given our partnership approach to working with schools and their students as true research partners as per CBPR principles [49]–especially relational accountability and mindful reciprocity to equalize the power imbalances between the academic community and Indigenous communities [50]. In terms of compliance and response rate, there were attendance concerns with some of the youth which impacted the follow-up focus group for boys. While using focus group data alone can be seen as a possible limitation, this study is part of the larger Smart Platform initiative, which uses interviews as well as mixed-methods data related to smartphone usage [51], youth mental health [52], and mobile health [53]. Thus, it is important to note that the findings of this study should be interpreted by taking into context the overall findings of the Smart Platform [21, 22, 48]. Another limitation includes logistical issues, as having a quiet space in a busy K-12 school to conduct the focus groups was challenging. Technological challenges included inconsistent WiFi access, which required creative solutions from the researchers such as convincing schools to provide uninterrupted WIFI access to participating citizen scientists before, during, and after school hours. Students without consistent Internet access at home may have been excluded from participating fully in the SIY project. Lastly, the self-reporting component of the SIY project increases the bias and burden on participants.

## 5. Conclusions

This novel qualitative quasi-experimental study offers a window into the future of upstream interventions in partnership with Indigenous communities, where Indigenous youth can be engaged in real-time via their digital devices, while participating in culturally-sensitive, land-based school programming that promotes culture, identity, and mental health. Culturally-appropriate, land-based, active living school programs have immense potential to benefit Indigenous students by increasing school attendance, and enabling equitable access to upstream mental health solutions. Due to historical trauma and losses through the residential schools and other assimilationist measures, not all Indigenous families have cultural knowledge or access to cultural teachers; therefore, providing these programs through schools creates an equalizing dynamic to cultural learnings that can be of great benefit to Indigenous youth. Indigenous communities know what is best when it comes to their youth and solutions for their youth need to be Indigenous-led and based in traditional value systems.

## Supporting information

S1 File Inclusivity in global research. (DOCX)

S1 Appendix (DOCX)

## List of abbreviations

SIY: Smart Indigenous Youth
TRC: Truth and Reconciliation Commission
YCSs: Youth Citizen Scientists

## References

[1] Allan B, Smylie J, Wellesley Institute, issuing body, Canadian Electronic Library, distributor. First peoples, second class treatment: the role of racism in the health and well-being of Indigenous peoples in Canada. 2015.

[2] Indigenous Foundations [Internet]. UBC First Nations and Indige nous Studies: 2009. Reserves; 2009 [cited 2021 Jan 29]. Available from https://indigenousfoundations.arts.ubc.ca/reserves/

[3] Health Canada [Internet]. Ottawa: Health Canada; 2013. National Aboriginal Youth Suicide Prevention Strategy (NAYSPS) Program Framework; 2013 [cited 2021 Jan 29]. Available from: https://www.canada.ca/content/dam/hc-sc/migration/hc-sc/fniah-spnia/alt_formats/pdf/pubs/promotion/_suicide/strat-prev-youth-jeunes-eng.pdf

[4] Centre for Suicide Prevention [Internet]. Centre for Suicide Prevention; 2013. Canada’s Indigenous Communities and Suicide; 2013 [cited 2020 Oct 10]. Available from: https://www.suicideinfo.ca/local_resource/indigenoussuicide/

[5] Evans W, Davis B. Exploring the Relationship between Sense of Coherence and Historical Trauma among American Indian Youth. American Indian & Alaska Native Mental Health Research: The Journal of the National Center. 2018 Oct 1;25(3). doi: 10.5820/aian.2503.2018.1 30320874

[6] Totten M. Aboriginal youth and violent gang involvement in Canada: Quality prevention strategies. IPC Review. 2009 Mar;3(1):135–56.

[7] Gates M, Hanning R, Gates A, Stephen J, Fehst A, Tsuji L. Physical activity and fitness of First Nations youth in a remote and isolated northern Ontario community: a needs assessment. Journal of community health. 2016 Feb;41:46–56. doi: 10.1007/s10900-015-0063-8 26175076

[8] Patel V, Flisher AJ, Hetrick S, McGorry P. Mental health of young people: a global public-health challenge. The Lancet. 2007 Apr 14;369(9569):1302–13. doi: 10.1016/S0140-6736(07)60368-7 17434406

[9] Malla A, Boksa P, Joober R. The new wave of youth mental health services: time for reflection and caution. The Canadian Journal of Psychiatry. 2021 Jul;66(7):616–20. doi: 10.1177/0706743720984382 33375848 PMC8240002

[10] Call KT, Riedel AA, Hein K, McLoyd V, Petersen A, Kipke M. Adolescent health and well‐being in the twenty‐first century: a global perspective. Journal of research on adolescence. 2002 Mar;12(1):69–98.

[11] Centre for Suicide Prevention [Internet]. Centre for Suicide Prevention; 2018. Saskatchewan First Nations Suicide Prevention Strategy; 2018 [cited 2020 Oct 10] Available from https://www.suicideinfo.ca/wp-content/uploads/gravity_forms/6-191a85f36ce9e20de2e2fa3869197735/2018/07/Saskatchewan-First-Nations-Suicide-Prevention-Strategy_oa.pdf

[12] CBC [Internet]. CBC; 2016. La Loche hopes for a better future after deadly shootings, struggles with youth suicides; 2016 January [cited 2020 Oct 10]. Available from: https://www.cbc.ca/news/canada/saskatchewan/la-loche-monday-matthew-kruchak-youth-suicide-mental-health-resources-1.3418191

[13] Aguiar W, Halseth R. Aboriginal peoples and historic trauma: the processes of intergenerational transmission. 2015. Prince George, BC: National Collaborating Centre for Aboriginal Health.:32.

[14] Snowshoe A, Crooks CV, Tremblay PF, Hinson RE. Cultural connectedness and its relation to mental wellness for First Nations youth. The Journal of Primary Prevention. 2017 Apr;38:67–86. doi: 10.1007/s10935-016-0454-3 27807659

[15] Ritchie SD, Wabano MJ, Russell K, Enosse L, Young NL. Promoting resilience and wellbeing through an outdoor intervention designed for Aboriginal adolescents. Rural and remote health. 2014 Jan 1;14(1):83–101. 24670144

[16] Dockery AM. Inter-generational transmission of Indigenous culture and children’s wellbeing: Evidence from Australia. International Journal of Intercultural Relations. 2020 Jan 1;74:80–93.

[17] Davison CM, Hawe P. School engagement among aboriginal students in Northern Canada: perspectives from activity settings theory. Journal of School Health. 2012 Feb;82(2):65–74. doi: 10.1111/j.1746-1561.2011.00668.x 22239131

[18] Coppola AM, McHugh TL. Considering culturally relevant practices and knowledge-sharing when creating an activity-promoting community research agenda. Sport, education and society. 2018 Jan 2;23(1):14–27.

[19] Dubnewick M, Hopper T, Spence JC, McHugh TL. “There’s a cultural pride through our games”: Enhancing the sport experiences of Indigenous youth in Canada through participation in traditional games. Journal of Sport and Social Issues. 2018 Aug;42(4):207–26.

[20] Clark N, Drolet J. “Melq’ilwiye” Coming Together: Reflections on the journey towards Indigenous social work field education. Currents: Scholarship in the Human Services. 2014;13(1).

[21] Katapally TR. The SMART framework: Integration of citizen science, community-based participatory research, and systems science for population health science in the digital age. JMIR mHealth and uHealth. 2019 Aug 30;7(8):e14056. doi: 10.2196/14056 31471963 PMC6743262

[22] Katapally TR. Smart indigenous youth: The smart platform policy solution for systems integration to address indigenous youth mental health. JMIR Pediatrics and Parenting. 2020 Sep 25;3(2):e21155. doi: 10.2196/21155 32975527 PMC7547388

[23] Martin DH. Two-eyed seeing: a framework for understanding indigenous and non-indigenous approaches to indigenous health research. Canadian Journal of Nursing Research Archive. 2012 Jun 1:20–43. 22894005

[24] Coppola AM, Holt NL, McHugh TL. Supporting Indigenous youth activity programmes: A community-based participatory research approach. Qualitative Research in Sport, Exercise and Health. 2020 May 26;12(3):319–35.

[25] Hennink M, Kaiser BN. Sample sizes for saturation in qualitative research: A systematic review of empirical tests. Social Science & Medicine. 2022 Jan 1;292:114523. Sample sizes for saturation in qualitative research: A systematic review of empirical tests—ScienceDirect doi: 10.1016/j.socscimed.2021.114523 34785096

[26] Guest G, Namey E, Chen M. A simple method to assess and report thematic saturation in qualitative research. PloS one. 2020 May 5;15(5):e0232076. A simple method to assess and report thematic saturation in qualitative research | PLOS ONE 32369511 10.1371/journal.pone.0232076 PMC7200005

[27] Mishra L. Focus group discussion in qualitative research. TechnoLearn: An International Journal of Educational Technology. 2016;6(1):1–5. https://www.indianjournals.com/ijor.aspx?target=ijor:tle&volume=6&issue=1&article=001

[28] Mason M. Sample size and saturation in PhD studies using qualitative interviews. InForum qualitative Sozialforschung/Forum: qualitative social research 2010 Aug 24 (Vol. 11, No. 3). Sample Size and Saturation in PhD Studies Using Qualitative Interviews | Forum Qualitative Sozialforschung / Forum: Qualitative Social Research (qualitative-research.net)

[29] First Nations Information Governance Committee [Internet]. Ottawa: National Aboriginal Health Organization; 2007. OCAP: Ownership, Control, Access and Possession; 2007 [cited 2021 Jan 29]. Available from: www.fnigc.ca

[30] Government of Canada Panel on Research Ethics [Internet]. Ottawa: Interagency Advisory Panel on Research Ethics; 2023. TCPS 2 (2018)–Chapter 9: Research Involving the First Nations, Inuit and Métis Peoples of Canada; 2018 [updated 2022; cited 2021 Jan 29]. Available from: https://ethics.gc.ca/eng/tcps2-eptc2_2018_chapter9-chapitre9.htm

[31] Canadian Institute for Health Research (CIHR) [Internet]. Ottawa: Canadian Institute for Health Research; 2020. Defining Indigenous Health Research; 2020 [updated 2023; cited 2021 Jan 29]. Available from: https://irsc-cihr.gc.ca/e/50340.html

[32] First Nations Health Authority (FNHA) [Internet]. What is Land-Based Treatment and Healing. [n.d., cited May 3, 2023]. Available from:FNHA-What-is-Land-Based-Treatment-and-Healing.pdf

[33] Wexler L. The importance of identity, history, and culture in the wellbeing of indigenous youth. The Journal of the History of Childhood and Youth. 2009;2(2):267–76. 10.1353/hcy.0.0055

[34] MacDonald JP, Ford JD, Willox AC, Ross NA. A review of protective factors and causal mechanisms that enhance the mental health of Indigenous Circumpolar youth. International journal of circumpolar health. 2013 Jan 31;72(1):21775. doi: 10.3402/ijch.v72i0.21775 24350066 PMC3860333

[35] Kirmayer LJ, Sheiner E, Geoffroy D. Mental health promotion for indigenous youth. In Positive mental health, fighting stigma and promoting resiliency for children and adolescents 2016 Jan 1 (pp. 111–140). Academic Press. 10.1016/B978-0-12-804394-3.00006-1

[36] Absolon K. Indigenous wholistic theory: A knowledge set for practice. First peoples child & family review. 2020 May 5;5(2):74–87.

[37] Baikie G. Indigenous-centered social work: Theorizing a social work way-of-being. Wicihitowin: Aboriginal social work in Canada. 2009:42–61.

[38] Gray M, Bird MY, Coates J. Towards an understanding of indigenous Social work. InIndigenous social work around the world 2016 May 23 (pp. 77–86). Routledge.

[39] Loiselle M, McKenzie L. The wellness wheel: An Aboriginal contribution to social work. Noranda: Université du Québec en Abitibi-Témiscamingue; 2006 May 27.

[40] Simpson L, McFadden M, Munns G. Someone has to go through’: Indigenous boys, staying on at school and negotiating masculinities. What about the boys. 2001 Oct 1:154–68.

[41] Henry R. Through an Indigenous Lens: Understanding Indigenous Masculinity and Street Gang Involvement.

[42] Anderson K, Innes R, Swift J. Indigenous masculinities: Carrying the bones of the ancestors. Canadian men and masculinities: Historical and contemporary perspectives. 2012:266–84.

[43] Walsh R, Danto D, Sommerfeld J. Land-based intervention: A qualitative study of the knowledge and practices associated with one approach to mental health in a Cree community. International Journal of Mental Health and Addiction. 2020 Feb;18:207–21.

[44] Aboriginal Peoples’ Television Network [Internet]. APTN; 2016. Saskatchewan elementary school offers Cree immersion to students; 2016 April 29 [cited 2020 Oct 10]. Available from: https://www.aptnnews.ca/national-news/saskatchewan-elementary-school-offers-cree-immersion-to-students/

[45] CBC [Internet]. CBC; 2019. Land-based education in Sask. Education system not just a trend: teachers; 2019 May 30 [cited 2020 Oct 10]. Available from: https://www.cbc.ca/news/canada/saskatchewan/land-based-education-more-than-a-trend-1.5156457

[46] Ross JB. Indigenous intergenerational teachings: The transfer of culture, language, and knowledge in an intergenerational summer camp. American Indian Quarterly. 2016 Jul 1;40(3):216–50.

[47] Yang T, Warburton DE. Indigenous elders’ role in fostering intergenerational relationships with youth. The Health & Fitness Journal of Canada. 2018 Dec 30;11(4):88–93.

[48] Katapally TR, Bhawra J, Leatherdale ST, Ferguson L, Longo J, Rainham D, et al. The SMART study, a mobile health and citizen science methodological platform for active living surveillance, integrated knowledge translation, and policy interventions: longitudinal study. JMIR Public Health and Surveillance. 2018 Mar 27;4(1):e8953. doi: 10.2196/publichealth.8953 29588267 PMC5893892

[49] Koster R, Baccar K, Lemelin RH. Moving from research ON, to research WITH and FOR Indigenous communities: A critical reflection on community‐based participatory research. The Canadian Geographer/Le Géographe canadien. 2012 Jun;56(2):195–210.

[50] Tobias JK, Richmond CA, Luginaah I. Community-based participatory research (CBPR) with indigenous communities: producing respectful and reciprocal research. Journal of Empirical Research on Human Research Ethics. 2013 Apr;8(2):129–40. doi: 10.1525/jer.2013.8.2.129 23651937

[51] Brodersen K, Hammami N, Katapally TR. Smartphone use and mental health among youth: it is time to develop smartphone-specific screen time guidelines. Youth. 2022 Feb 7;2(1):23–38.

[52] Kannan P, Bhawra J, Patel P, Katapally TR. Preserving rural school health during the COVID-19 pandemic: Indigenous citizen scientist perspectives from a qualitative study. AIMS public health. 2022;9(2):216. doi: 10.3934/publichealth.2022016 35634029 PMC9114787

[53] Katapally TR, Hammami N, Chu LM. A randomized community trial to advance digital epidemiological and mHealth citizen scientist compliance: A smart platform study. Plos one. 2021 Nov 1;16(11):e0259486. doi: 10.1371/journal.pone.0259486 34723987 PMC8559921

