# Supplementary material for: Evaluation of a longitudinal digital citizen science initiative to understand the impact of culture on Indigenous youth mental health: Findings from a quasi-experimental qualitative study
Source: PLoS One. 2023 Dec 21;18(12):e0294234. doi: 10.1371/journal.pone.0294234 (PMC10735025; doi:10.1371/journal.pone.0294234)
Supplement: S1 Appendix — (DOCX) [file pone.0294234.s002.docx]

**Appendix 1**

**Smart Indigenous Youth (SIY) Focus Group 2**

**Culture and identity**

*The first few questions we’ll discuss are about your culture and identity. Culture means where you’re from, teachings from your family and community, and our history.*

1a) What kind of cultural teachings/activities were part of your school program?

1b) What did you learn about your culture from the school program?

2) Has learning about your culture become more important to you after participating in this program?

3a) Do you feel a change in your cultural identity since starting the program?

3b) What did you learn about your cultural identity? Do you feel a stronger connection to your culture since starting the school program?

*Transition (if possible). Otherwise note that we’re moving to another topic area (i.e., now we’d like to talk about physical activity in your community).*

**Active Living**

4) What kind of activities did you learn and participate in?

5) What kind of cultural or traditional activities did you participate in as a result of this program?

6) Do you think the program helped you become more culturally involved?

*Transition into next topic by noting that physical activity is known to affect your mental health and how you feel.*

**Mental Health**

7) Do you feel that the program affected your mental health in any way?

Probe: Have there been any changes to your mental health as a result of participating in the program?

8) How do you feel about being in school as a result of this program?
